# Platelet indices significantly correlate with liver fibrosis in HCV-infected patients

**DOI:** 10.1371/journal.pone.0227544

**Published:** 2020-01-09

**Authors:** Lin-Nan Shao, Shu-Ting Zhang, Ni Wang, Wei-Jian Yu, Mei Chen, Nan Xiao, Ying Duan, Ling-Zi Pan, Wen-Qian Song, Yue-Xin Xia, Li Zhang, Ning Qi, Ming Liu, Shi-Hang Zhou

**Affiliations:** 1 Dalian Blood Center, Zhongshan District, Dalian, Liaoning, China; 2 Department of Cell Biology, Dalian Medical University, Dalian, Liaoning, China; Centre de Recherche en Cancerologie de Lyon, FRANCE

## Abstract

**Aim:**

A total of 241 patients with chronic HCV infection were recruited to investigate the association between liver fibrosis and PLT counts, as well as with MPV, PDW and P-LCR indices.

**Methods:**

The determination of PLT indices was carried out using a Sysmex XT-1800i automated hematology analyzer. Serological tests for HA, LN, C-IV and PIIINP were performed in 210 patients. The liver stiffness was measured in 69 patients by transient elastography (FibroScan).

**Results:**

The analysis showed that the four serum fibrosis markers were negatively correlated with PLT counts, but positively correlated with the MPV, PDW and P-LCR values. Moreover, a similar pattern was found after analyzing the FibroScan measurements, which were negatively correlated with PLT counts, but positively correlated with MPV, PDW and P-LCR values. We subdivided the HCV-infected patients into mild and advanced fibrosis groups. The PLT counts were significantly decreased and the MPV, PDW and P-LCR values were significantly increased in the advanced fibrosis group when compared with the mild fibrosis group.

**Conclusions:**

Our results demonstrate that not only the PLT counts but also the MPV, PDW and P-LCR indices significantly correlate with liver fibrosis in HCV-infected patients. Therefore, these indices may be useful laboratory measures for evaluating liver fibrosis progression.

## Introduction

Hepatitis C virus (HCV) infection is one of the most important causes of chronic liver disease, affecting approximately 3% of the population worldwide [1]. Most individuals with chronic HCV infection will develop hepatic fibrosis, cirrhosis and complications of end-stage liver disease [2]. Fibrosis progression is an early indicator of disease severity in patients with chronic HCV infection. In recent years, several noninvasive methods have been used to determine the extent of liver fibrosis in chronic liver diseases. These include serum-based biomarkers like hyaluronic acid (HA), laminin (LN), collagen IV (C-IV) and amino-terminal pro-peptide of Type-III pro-collagen (PIIINP), as well as transient elastography (FibroScan®) [3, 4].

**Fig 1:**
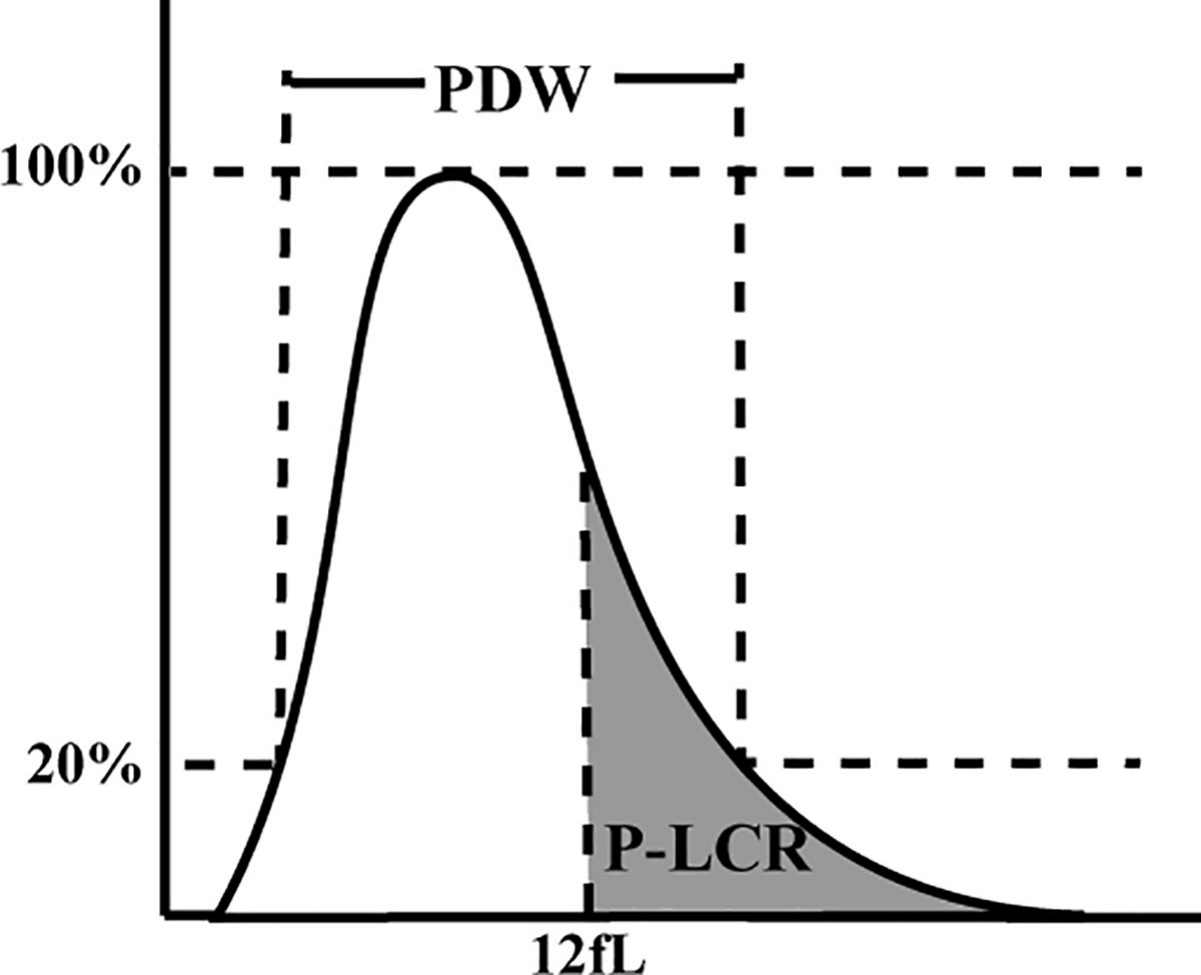
Platelet histogram. The distribution width at the level of 20% was defined as platelet distribution width (PDW), and the percentage of the platelets with a size of more than 12 fL was defined as platelet-large cell ratio (P-LCR).

The platelet (PLT) count, mean platelet volume (MPV), platelet distribution width (PDW) and platelet large cell ratio (P-LCR) are routine parameters analyzed in the complete blood count test. These PLT indices can be easily measured by using full blood count analyzers and their measurement is cost-effective. MPV describes the average PLT size reported in femtolitres (fL). PDW is defined as the distribution width at a 20% frequency level (the peak of the histogram is 100%) and is a measure of PLT size heterogeneity. P-LCR is the percentage of PLTs with a size of more than 12 fL (Fig 1). The PLT count has been shown to be an important severity index in several diseases, such as malaria [5], colorectal cancer [6] and acute kidney injury [7]. It is noteworthy that the PLT count is also a convenient marker of liver fibrosis in several hepatic diseases, such as nonalcoholic fatty liver disease [8], hepatitis B [9] and C [10]. Nevertheless, there are studies reporting conflicting results [11, 12]. In addition, the MPV, PDW and P-LCR indices are generally ignored by doctors and data on MPV, PDW and P-LCR and liver fibrosis are limited. Thus, the aim of this study was to investigate the association between liver fibrosis and the PLT count, as well as the MPV, PDW and P-LCR indices in HCV-infected patients.

## Methods

### Patients and samples

A total of 241 patients with chronic HCV infection were recruited from Dalian Infectious Hospital from April to December 2015. None of the patients had received previous treatments with antiviral drugs. To avoid introducing bias through sample selection, our inclusion criterium was HCV infection, whereas the exclusion criteria were presence of ascites, concurrent hepatitis B virus infection, alcohol-related liver disease, significant pre-existing organ (heart, brain, lung or kidney) complications and presence of diseases that could affect PLTs such as atherosclerotic diseases, rheumatic diseases, hematologic disorders, etc. A fasting venous blood sample was collected from each patient in a vacutainer tube containing ethylenediaminetetraacetic acid. This work was conducted in compliance with the ethical principles of the Helsinki Declaration. All data were anonymized to comply with the provisions of personal data protection legislation. Informed consent was obtained from all participants. This study was approved by the Dalian Blood Center Ethics Committee (No: DBC00802009).

### Serological tests

All blood samples were collected, handled and processed in the same way at room temperature (about 22°C). The determination of PLT indices was carried out within one hour of sample collection using an automated hematology analyzer (XT-1800i, Sysmex Corporation, Kobe, Japan). The detection limits of PLT counts were 0–9999 (×10^9^/L). The coefficients of variation of PLT, MPV, PDW and P-LCR were ≤ 4.0%, 4.0%, 10.0% and 18.0%, respectively. Autoagglutinated and hemolytic samples cannot be used. The HA, LN, C-IV and PIIINP serological tests of 210 patients were performed by Autobio Diagnostics Co., Ltd. (Zhengzhou, Henan Province, China). All procedures were performed following the manufacturer’s instructions.

### FibroScan® measurements

Sixty-nine patients had liver stiffness, based on transient elastography (FibroScan®, Echosens, Paris, France). At least 10 valid measurements with a success rate of at least 60%, and an interquartile range of less than 30% of the median elasticity were considered successful. The liver stiffness values relate to the validated liver fibrosis METAVIR fibrosis stages with the following cutoff values [13]: F0-1<7.1 kPa; F2 > or = 7.1 kPa; F3> or = 9.6 kPa; F4> or = 11.6 kPa.

### Statistical analysis

The Kolmogorov-Smirnov test was used to analyze whether the data showed a Gaussian distribution. If a Gaussian distribution was confirmed, the results were expressed as the mean ± standard deviation and compared using Student’s *t*-test. In the absence of a Gaussian distribution, findings were expressed as the median (interquartile range) and analyzed using the Mann-Whitney *U*-test. Correlations between PLT indices and liver fibrosis severity were analyzed using Spearman's rank correlation coefficient. A *P*-value of less than 0.05 was considered statistically significant. Statistical analyses were performed using SPSS version 21.0.

## Results

**Fig 2:**
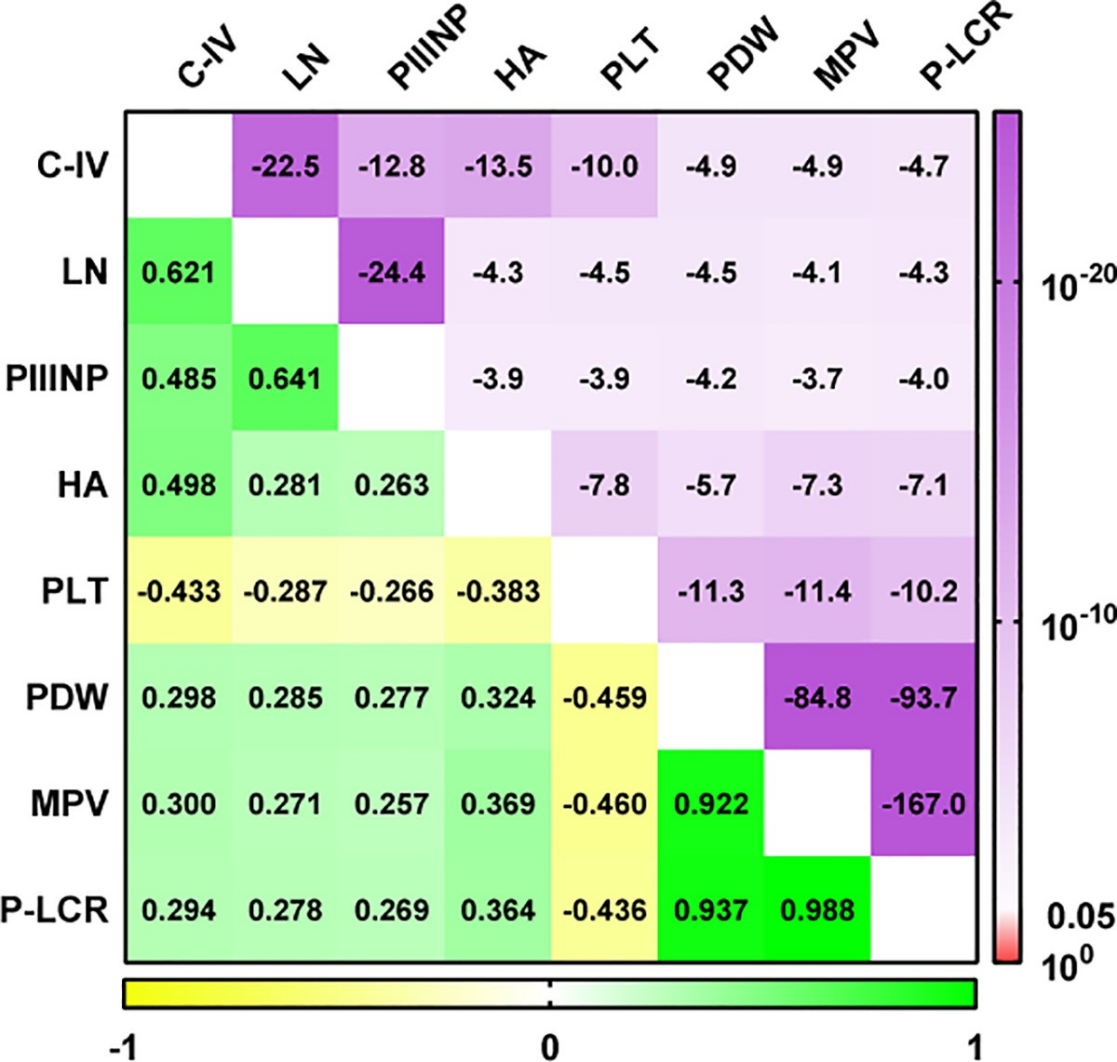
Spearman's rank correlations between platelet indices and four serum fibrosis markers. The upper triangle represents the P values and the lower triangle represents the rho values. C-IV, collagen IV; LN, laminin; PIIINP, amino-terminal pro-peptide of Type-III pro-collagen; HA, hyaluronic acid; PLT, platelet; PDW, platelet distribution width; MPV, mean platelet volume; P-LCR, platelet large cell ratio.

**Fig 3:**
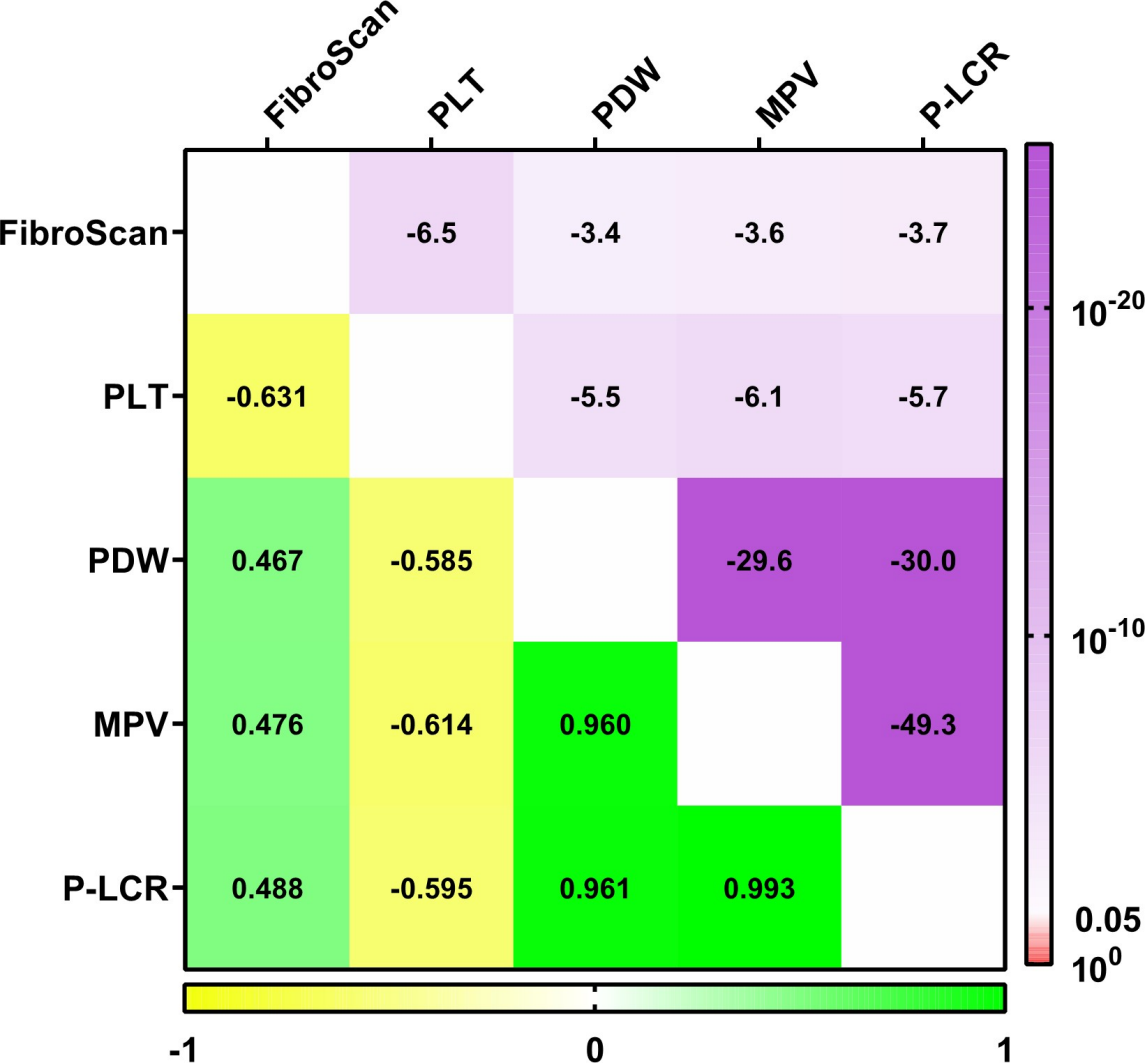
Spearman's rank correlations between PLT indices and FibroScan measurements. The upper triangle represents the P values and the lower triangle represents the rho values. PLT, platelet; PDW, platelet distribution width; MPV, mean platelet volume; P-LCR, platelet large cell ratio.

**Fig 4:**
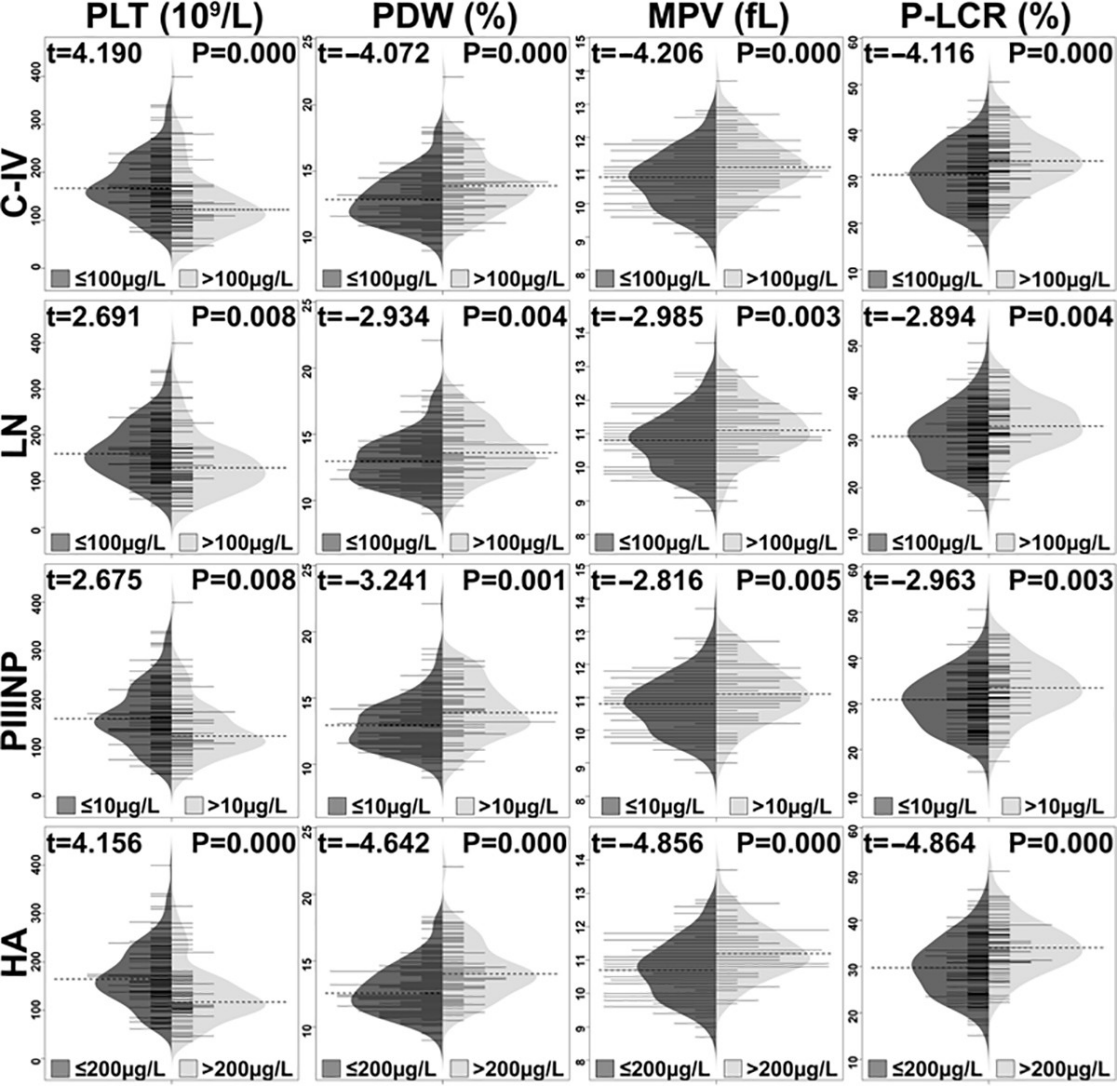
The comparison of PLT indices between mild and advanced fibrosis groups. C-IV, collagen IV; LN, laminin; PIIINP, amino-terminal pro-peptide of Type-III pro-collagen; HA, hyaluronic acid; PLT, platelet; PDW, platelet distribution width; MPV, mean platelet volume; P-LCR, platelet large cell ratio.

The demographic and laboratory characteristics of the patients are listed in Table 1. Correlations between the PLT indices and the four serum markers are shown in Fig 2. Our analysis showed that the four serum fibrosis markers were negatively correlated with the PLT counts, and positively correlated with the MPV, PDW and P-LCR values. Of the four fibrosis markers, C-IV showed the strongest and most inverse correlation with PLT counts (rho = -0.433, *P* = 10^−10.0^), while HA showed the strongest and most positive correlation with PDW, MPV and P-LCR values (rho = 0.324, *P* = 10^−5.7^, rho = 0.369, *P* = 10^−7.3^ and rho = 0.364, *P* = 10^−7.1^, respectively). Correlations between PLT indices and FibroScan® measurements are shown in Fig 3. A similar pattern was found, i.e., FibroScan® measurements were correlated negatively with PLT counts, but positively correlated with MPV, PDW and P-LCR values. Figs 2 and 3 illustrated that MPV, PDW and P-LCR were strong positive correlation with each other and negative correlation with PLT counts. Additionally, we subdivided the HCV-infected patients into mild and advanced fibrosis groups, based on the serum fibrosis indices (demarcations for C-IV, LN, PIIINP and HA were 100 μg/L, 100 μg/L, 10 μg/L and 200 μg/L, respectively), and then used beanplots [14] to compare the PLT indices between the two groups. The results are shown in Fig 4. Moreover, according to the FibroScan® measurements, we found that the numbers of patients for four fibrosis stage groups (F1, F2, F3 and F4) were 24, 13, 11 and 21, respectively. The differences between platelet indices in patients with mild (F1, F2) and advance fibrosis (F3, F4) were shown in Table 2. Both Fig 4 and Table 2 indicated that the PLT counts were significantly decreased and the MPV, PDW and P-LCR values were significantly increased in the advanced fibrosis groups, when compared with the mild fibrosis groups (all *P*-values<0.05).

**Table 1:** Demographic and laboratory features of the HCV patients.

		n	HCV patients
Age	year	241	53.7±13.8
Gender	M/F	241	127/114
Platelet	10^9^/L	241	157.1±63.7
MPV	fL	241	10.9±0.9
PDW	%	241	13.4±2.1
P-LCR	%	241	31.5±6.5
FibroScan	kPa	69	8.9(6.1–13.9)
C-IV	μg/L	210	85.5(65–133.5)
LN	μg/L	210	73.5(44–110.75)
PIIINP	μg/L	210	3(3–12.75)
HA	μg/L	210	134(77.25–224.5)

C-IV, collagen IV; HA, hyaluronic acid; HCV, Hepatitis C virus; LN, laminin; MPV, mean platelet volume; PIIINP, amino-terminal pro-peptide of Type-III pro-collagen; PDW, platelet distribution width; P-LCR, platelet large cell ratio.

**Table 2:** The differences between platelet indices in patients with mild (F1, F2) and advance fibrosis (F3, F4).

		PLT (10^9^/L)	PDW (%)	MPV (fL)	P-LCR (%)
Group	F1, F2	178.56±54.14	12.65±1.44	10.60±0.73	29.12±5.09
	F3, F4	120.06±65.53	14.15±2.62	11.23±0.95	33.87±7.04
*P*		0.000	0.007	0.003	0.002

MPV, mean platelet volume; PDW, platelet distribution width; P-LCR, platelet large cell ratio; PLT, platelet.

## Discussion

Various blood cell parameters can be measured by automated blood cell analyzers. Among these parameters, the MPV, PDW, and P-LCR indices provide important information but are generally ignored or not fully accepted in routine clinical practice. Whether the PLT indices are clinically useful laboratory tests of liver fibrosis in patients with HCV has not been fully established.

In this study, we found that liver fibrosis in HCV-positive patients was negatively correlated with the PLT counts, and positively correlated with the MPV, PDW and P-LCR values. Moreover, patients with advanced liver fibrosis had significantly lower PLT counts and significantly higher MPV, PDW and P-LCR values when compared with patients with mild fibrosis. These findings indicate that the PLT count and MPV, PDW and P-LCR indices can be used as surrogate markers of fibrosis progression or regression in HCV-infected patients.

Several reports indicate that thrombocytopenia is one of the most common hematological problems found in chronic HCV-infected patients [15–17]. Moreover, the PLT count is a convenient marker of liver fibrosis in HCV-infected patients [10, 18] and has been included in several indices, such as APRI and FIB-4, for the noninvasive prediction of the severity of hepatic fibrosis. The mechanism underlying the reduced PLT counts in patients with severe fibrosis has been attributed to various processes, such as reduced hepatic production of thrombopoietin, increased splenic sequestration and destruction of PLTs in the enlarged spleen secondary to the myelosuppressive action of HCV, or portal hypertension [19, 20]. In addition, it has been demonstrated that a low PLT count is predictive of the development of hepatocellular carcinoma [21, 22]. Thus, maintaining a PLT count above the normal level may be favorable during the management of chronically infected HCV patients [23]. Due to enhanced breakdown of PLTs in the spleen and increased interleukin-6 levels, the PLT life cycle is shorter in patients with chronic liver diseases. This stimulates PLT production by the bone marrow and promotes the release of larger, reticulated PLTs into the bloodstream. This increased entry of PLTs will result in increased MPV, PDW and P-LCR values. Our results also showed that fibrosis progression was positively correlated with MPV, PDW and P-LCR values. Moreover, MPV, PDW and P-LCR were strongly correlated with each other. Purnak et al. [11] reported that MPV increased with advanced fibrosis in HCV patients. However, our study has several advantages: (1) a larger sample size; (2) the inclusion of two noninvasive methods, serum-based biomarkers and transient elastography to assess liver fibrosis; and (3) additional PLT indices (PDW and P-LCR) that were positively associated with fibrosis progression. Interestingly, an HIV/HCV coinfection study [24] demonstrated that although both HIV and HCV viruses were associated with reduced PLT counts, the MPV did not change significantly in coinfected patients. The reason was that although HCV infection increased the MPV, HIV infection decreased this index and these two opposing trends counteracted each other during HIV/HCV coinfection. This suggests that the MPV can either decrease or increase in pathological conditions [25]. This study has also some limitations as lack of evaluation of dynamics of serum fibrosis markers and PLT indices, and relatively small sample size.

In summary, we demonstrated that not only the PLT count but also the MPV, PDW and P-LCR indices were significantly correlated with liver fibrosis in HCV-infected patients. These indices may provide laboratory measures for evaluating the progression of liver fibrosis but further research is necessary to validate their clinical application.

## Supporting information

S1 File Anonymized study dataset. (XLSX)
